# Supplementary material for: Influence of Lipopolysaccharide-Interacting Peptides Fusion with Endolysin LysECD7 and Fatty Acid Derivatization on the Efficacy against Acinetobacter baumannii Infection In Vitro and In Vivo
Source: Viruses. 2024 May 11;16(5):760. doi: 10.3390/v16050760 (PMC11125741; doi:10.3390/v16050760)
Supplement: Supplementary file 1 [file viruses-16-00760-s001.zip › viruses-2945646-supplementary.pdf]

Supplementary Information

Influence of LPS-interacting peptides fusion with endolysin LysECD7 and fatty acid derivatization on the efficiency against *Acinetobacter baumannii in vitro* and *in vivo*

Xiaowan Li[1]· Wenwen Shangguan[1]·Xiaoyue Hu[2]·Meiqing Feng[1*]·Jun Feng[2*]·Wenjie Zhao[1,2*]

[1] School of pharmacy, Fudan University, Shanghai, China, 201203

[2] China State Institute of Pharmaceutical Industry, Shanghai, China, 201203

Supplementary Table S1. Sequences of LPS-interacting peptides.

peptide	sequence
Li5	KNYSSSISSIRA
MSI594	GIGKFLKKAKKGIGAVLKVLTTG
Li5-MSI594	KNYSSSISSIRAGGGGSGGGGSGGGGSGIGKFLKKAKKGIGAVLKVLTTG

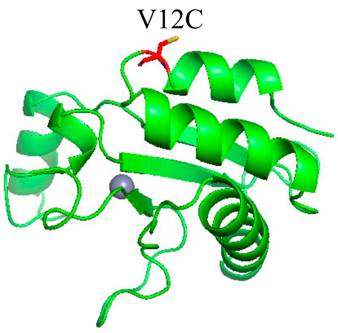

Figure S1. The predicted three-dimensional structure of LysECD7 (V12C).

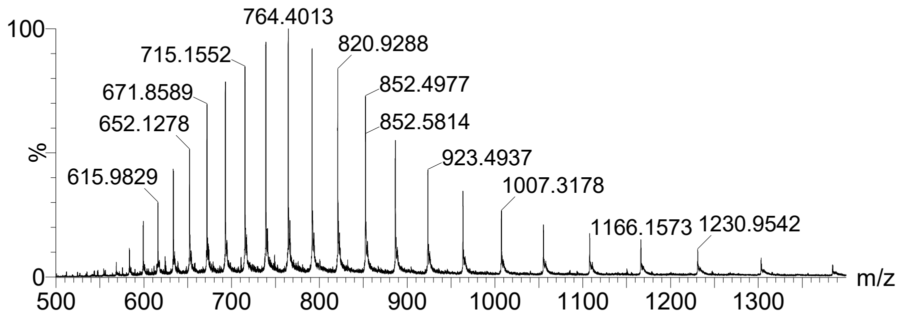

Figure S2. ESI-MS of V12C-C16.
